# Single‐Cell and Bulk Transcriptomic Profiling of Urothelial Carcinoma: Immunotherapy Associations and Functional Characterization of LY6E

**DOI:** 10.1155/ijog/8444243

**Published:** 2026-11-11

**Authors:** Yitao Huang, Chao Zhang, Xiuzhuang Xie, Xiaye Miao, Zhang Quan

**Affiliations:** ^1^ Department of Urology Yongkang Hospital of Traditional Chinese Medicine Affiliated to Zhejiang University of Chinese Medicine Yongkang China dlut.edu.cn; ^2^ Central Laboratory Precision Medicine Center Affiliated Jinhua Hospital Zhejiang University School of Medicine Jinhua China zju.edu.cn; ^3^ Department of Laboratory Medicine Northern Jiangsu People′s Hospital Yangzhou China yzsbh.com; ^4^ Department of Traditional Chinese Medicine Oncology The Affiliated Huai′an Hospital of Xuzhou Medical University and the Second People′s Hospital of Huai′an Huai′an China

**Keywords:** bulk transcriptomics, immune checkpoint inhibition, LY6E, single-cell RNA sequencing, transcriptomic deconvolution, tumor immune microenvironment, urothelial carcinoma

## Abstract

**Background:**

Translating single‐cell immune profiles into bulk transcriptomic biomarkers requires distinguishing cell‐state signals from overall immune abundance. We evaluated the recovery and clinical relevance of single‐cell–derived immune modules in urothelial carcinoma and investigated LY6E as a candidate for functional follow‐up.

**Methods:**

We analyzed 73,279 single cells from two GSE223971 preparations, with eight matched CD45‐enriched single‐cell/bulk pairs and six broader preparation pairs from five patients. Immune modules underwent donor‐support filtering, patient‐level validation, lineage adjustment, and comparison with reference‐profile methods. Clinical associations were assessed in GSE176307 and IMvigor210, with repeated cross‐validation used to evaluate incremental prediction in IMvigor210. An expression‐based screen followed by clinical and immune‐context assessment prioritized LY6E for knockdown and rescue experiments in 5637 and J82 cells.

**Results:**

Thirteen of 39 annotated states met donor‐support criteria. Four modules passed the initial transfer screen, although all four 95% bootstrap intervals included zero; none of 11 testable subtypes met the within‐lineage specificity criteria. Plasma alone passed the cross‐preparation module screen (patient‐level Spearman rho = 0.900; exact *p* = 0.083), with weaker recovery by ProM (rho = 0.700). Its association with complete response versus progressive disease in the selected IMvigor210 subset attenuated after immune adjustment, and adding Plasma yielded no consistent predictive improvement. Several immune scores were associated with survival in unadjusted GSE176307 models without supported objective‐response associations. LY6E was upregulated in TCGA‐BLCA and GSE13507 tumors, but its associations with immunotherapy outcomes differed between cohorts. Two shRNAs reduced LY6E transcript abundance by 64%–67%, decreased growth and migration, and increased apoptosis in both cell lines. An shRNA‐resistant construct restored LY6E expression and clonogenic growth.

**Conclusions:**

Single‐cell and bulk transcriptomic integration recovered a Plasma‐related abundance signal without establishing subtype specificity or independent immunotherapy prediction. LY6E supported growth and survival in the tested bladder cancer cell lines, whereas its relevance as an immunotherapy biomarker remains unestablished.

## 1. Introduction

Bladder cancer remains a common cause of cancer morbidity and death, with marked variation in clinical course and treatment response [1]. Genomic studies have divided muscle‐invasive urothelial carcinoma into biologically distinct transcriptional groups [2], and the consensus molecular classification has made this heterogeneity easier to compare across cohorts [3]. Immune checkpoint inhibition can produce durable responses in advanced disease [4], but only a minority of patients benefit. Tumor‐extrinsic factors such as transforming growth factor‐beta–driven immune exclusion can limit response to programmed death‐ligand 1 blockade [5], whereas combined tissue and blood analyses indicate that treatment outcome is not captured by a single immune measurement [6]. A useful transcriptomic biomarker must therefore provide information beyond the broad immune context in which it is measured.

Single‐cell RNA sequencing (scRNA‐seq) offers a detailed view of that context. Bladder cancer studies have described inflammatory cancer‐associated fibroblasts [7], heterogeneous epithelial and immune populations [8], and coordinated multicellular ecosystems [9]. Tumor‐associated immune states have also been recovered from urine, providing a view across an anatomically connected compartment [10]. In checkpoint‐treated urothelial carcinoma, joint analysis of bulk and single‐cell data has linked myeloid programs to treatment resistance [11]. These studies expand the cellular vocabulary of the disease, whereas most cohorts with response and survival data remain available only as bulk expression profiles.

A single‐cell marker set can track the abundance of its source population in bulk RNA while providing little information about variation within that lineage. Its performance may also change with tissue composition. Evaluating these scores therefore requires matched measurements of cellular abundance, tests of within‐lineage specificity, and assessment across preparations. In clinical cohorts, the relevant question is whether a score adds information beyond overall immune content and other available covariates.

We evaluated these properties in urothelial carcinoma using exactly matched scRNA‐seq and bulk RNA profiles, a second single‐cell preparation with matched bulk data, and two immune checkpoint inhibitor cohorts. State markers were selected without reference to clinical outcomes and examined through resampling, patient‐level marker rederivation, parent‐lineage adjustment, alternative scoring rules, and reference‐profile methods. Clinical association was assessed separately from out‐of‐sample prediction. In a parallel analysis, an outcome‐independent expression and epithelial‐compatibility screen was followed by assessment of survival, external expression, and immune‐context evidence to prioritize LY6E. Knockdown and rescue experiments then tested its cell‐intrinsic effects in bladder cancer models.

## 2. Methods

### 2.1. Data Sources and Cohort Roles

Expression matrices, deposited analysis objects, and sample annotations were obtained from the Gene Expression Omnibus [12]. GSE223971 was the technical benchmark because it contained matched single‐cell and bulk RNA profiles from the same specimens. Its CD45‐enriched and compositionally broader objects comprised 43,827 and 29,452 cells from 10 nonoverlapping patients each. Exact single‐cell/bulk matches were available for eight CD45‐enriched patients and six broader preparation samples from five patients. GSE176307 provided normalized expression and clinical data for 89 patients with advanced urothelial carcinoma treated with immune checkpoint inhibitors, including 16 objective responders, 57 overall‐survival events among 89 patients, and 78 progression‐free‐survival events among 88 patients [13]. The easierData representation of IMvigor210 retained 192 of 348 expression‐profiled patients: 25 with complete response (CR) and 167 with progressive disease (PD), excluding 43 with partial response, 63 with stable disease, and 50 with unevaluable response. This analysis therefore compared CR with PD, not objective response across the full cohort; tumor mutational burden was available for 150 retained patients (21 CR and 129 PD).

Five additional datasets addressed predefined secondary questions rather than serving as direct replications of the matched benchmark. GSE176178 included 40 patients sampled before bacillus Calmette–Guérin treatment for high‐grade non–muscle‐invasive bladder cancer [14]. GSE244895 contained 35 longitudinal samples from 14 patients [15], whereas GSE199471 contained 28 longitudinal samples from 14 patients [16]. GSE145281 contributed peripheral‐blood single‐cell data from 10 checkpoint‐treated patients [17], and GSE269877 contributed 27 non–muscle‐invasive bladder cancer single‐cell samples from 23 patients [18]. TCGA‐BLCA and the curated Pan‐Cancer Clinical Data Resource supplied tumor expression and harmonized clinical endpoints for gene prioritization [19]. GSE13507 was used for independent expression and survival assessment [20], with the original E2F1 progression study providing cohort provenance [21].

### 2.2. Single‐Cell Object Processing and Quality Review

Analyses were performed in R 4.5.1. Public expression objects were handled with GEOquery 2.76.0, Biobase 2.68.0, and SummarizedExperiment 1.38.1, following established RNA‐seq preprocessing and identifier‐control principles [22]. The deposited GSE223971 objects were read with Seurat 5.4.0 and Matrix 1.7‐4. We retained the stored RNA data layer, original sample and patient identifiers, deposited major‐ and minor‐state annotations, and the supplied UMAP coordinates. The data structure followed the Seurat framework [23] and its later layer‐aware implementation [24]. Because the analysis tested transfer of the deposited cellular definitions, FASTQ‐level reprocessing, reintegration, reclustering, and expression‐based reannotation were not performed. Quality review was conducted separately for the 43,827‐cell CD45‐enriched object and the 29,452‐cell broader preparation object. For every sample and annotated lineage, we tabulated captured cells, unique molecular identifier counts, detected features, mitochondrial‐transcript percentage, and sample composition. Sample‐to‐patient links were reconciled with the bulk identifiers before any paired calculation. Donor contribution was evaluated for every minor state by patient count, maximum donor share, and normalized donor entropy. These checks covered the principal quality domains recommended for single‐cell analysis [25]. UMAP coordinates were used only to display the deposited structure and were not used to select cells, markers, or states.

### 2.3. Cell‐State Eligibility and Marker‐Module Derivation

The CD45‐enriched object contained 39 annotated minor states. A state entered the transfer benchmark only when it contained at least 100 cells, occurred in at least five patients, and received less than 40% of its cells from any single patient. These rules were applied jointly to reduce sparse‐state effects and prevent a nominal state signal from being driven by one donor. Thirteen states met all three criteria. State‐versus‐rest statistics were calculated with presto 1.0.0. Candidate genes required a difference of at least 0.25 between mean log‐normalized expression in the state and the remaining cells, and detection in at least 10% of cells assigned to the state. Genes were ranked by this expression difference, with adjusted *p* value as the secondary ordering criterion; the presto logFC field on this input scale was not a log2 fold change. Mitochondrial genes and ribosomal‐protein genes were removed, after which the 20 highest‐ranked eligible genes were retained as the state module. The same procedure was applied to the deposited major‐lineage labels to obtain parent‐lineage modules for specificity and clinical adjustment. Marker derivation used only the CD45‐enriched single‐cell object and did not use response, survival, or bulk‐expression information.

### 2.4. Bulk‐Expression Preprocessing and Module Scoring

GSE223971 and IMvigor210 transcript‐per‐million matrices were transformed as log2 (TPM+1). The author‐normalized GSE176307 matrix was analyzed on its deposited scale because raw counts were unavailable for uniform reprocessing. Gene identifiers were matched by exact symbol; the three primary bulk matrices contained no duplicated symbols. Coverage was recorded for the 13 state modules, and a primary state‐module score required at least five measured genes. Each predefined module was represented in bulk RNA by three outcome‐independent scoring rules. The z‐mean score was the mean of gene‐wise standardized expression values across samples. The rank‐mean score was the mean fractional rank of the module genes within each sample, providing a scale‐robust single‐sample summary [26]. The mean‐log score was the arithmetic mean of transformed expression across the measured module genes. These explicit scores were related to established single‐sample gene‐set scoring concepts [27] but were calculated directly rather than through an adaptive enrichment algorithm. Sensitivity to module length was assessed with the top 5, 10, 15, and 20 ranked genes.

### 2.5. Matched Single‐Cell‐to‐Bulk Transfer Benchmark

The primary technical benchmark used the eight independent CD45‐enriched pairs. For state s in sample i, the single‐cell truth was defined as (n_si+0.5)/(N_i+1), where n_si was the number of cells assigned to the state and N_i was the number of all captured cells in that sample. The pseudocount prevented undefined values in small states. Because dissociation, enrichment, and cell recovery affect the denominator, this quantity was termed the all‐captured‐cell fraction rather than whole‐tissue absolute abundance. A within‐parent fraction, (n_si+0.5)/(n_parent, i +1), was calculated separately for nonsingleton subtypes.

Spearman correlation quantified agreement between each bulk score and its matched all‐captured‐cell fraction. Pair‐level uncertainty was estimated from 5000 bootstrap resamples with replacement. Nonfinite bootstrap correlations were omitted without replacement before computing percentile limits. The 10th and 90th percentiles formed an exploratory screening interval, whereas the 2.5th and 97.5th percentiles formed the 95% uncertainty interval. Correlations were also recalculated after omitting each pair in turn. The initial transfer criteria required a 20‐gene z‐mean correlation of at least 0.50, correlations of at least 0.40 under all three scoring rules, a nested correlation of at least 0.50, and a positive lower screening‐interval bound. These correlation cutoffs and the 80% interval were study‐specific exploratory rules, not externally validated thresholds. A positive screening result did not imply significance at the 5% level when its 95% interval included zero.

### 2.6. Nested Marker Rederivation and Stability Analysis

For each of the eight matched patients, all single cells from that patient were excluded and markers were rederived from the other nine donors in the 10‐donor discovery object, retaining the fixed set of 13 eligible states. The same expression‐difference, detection, gene‐exclusion, and 20‐gene rules were used. Gene means and standard deviations for the held‐out bulk score were estimated only from the other seven matched bulk samples and then applied to the held‐out sample. Genes with zero training‐set variance contributed zero to the standardized score. The eight held‐out scores were correlated with their matched single‐cell fractions. This analysis assessed marker rederivation and scoring conditional on the fixed state‐eligibility rules, rather than independently validating the complete selection pipeline. Marker stability was summarized by the Jaccard overlap between each fold‐specific set and its full‐data module and by gene recurrence across folds. Sensitivity analyses crossed four module lengths with the three scoring rules, and leave‐one‐pair‐out ranges assessed dependence on individual specimens.

### 2.7. Parent‐Lineage Specificity Analysis

For each nonsingleton state, we asked whether the bulk module recovered variation within the parent lineage rather than merely tracking the amount of that lineage. The analysis compared six paired quantities: all‐captured‐cell state fraction, parent‐lineage fraction, within‐parent state fraction, raw state‐module score, the difference between state and parent‐module scores, and the residual from linear regression of the state score on the parent score. Parent modules were derived independently from the major‐lineage labels. Plasma and other singleton lineages had no within‐parent contrast and were therefore not evaluable for this comparison. A subtype was considered specific only when its raw state score correlated at least 0.50 with the within‐parent fraction and both the state‐minus‐parent score and parent‐adjusted residual correlated at least 0.40 with the same target. Correlation between the raw state and parent scores was reported to expose near‐duplicate representations. This matched‐sample estimand is different from between‐condition compositional testing, for which models such as scCODA address a separate question [28].

### 2.8. Cross‐Preparation Transportability Analysis

The broader GSE223971 preparation was used to test whether CD45‐derived modules remained informative after the observable cellular mixture changed. Marker lists, module lengths, scoring formulas, and decision thresholds were fixed before this analysis. Six matched single‐cell/bulk samples were available, but two samples belonged to the same patient; patient was therefore the independent unit. Single‐cell truth again used the all‐captured‐cell denominator. Sample‐level correlations were supplemented by a patient‐clustered bootstrap with 5000 resamples. Provisional support required a z‐mean correlation of at least 0.50, correlations of at least 0.40 under at least two of the three scoring rules, and a positive lower 80% clustered‐bootstrap bound. For the strict analysis, each sample was scored separately and its all‐captured‐cell fraction was calculated before the two scores and two fractions from the repeated patient were averaged with equal weight; cell counts were not pooled. Thereafter, the patient‐level z‐mean correlation had to be at least 0.50, and its exact two‐sided permutation *p* value had to be no greater than 0.10. With five patients, all 120 permutations of patient labels were enumerated.

### 2.9. Reference‐Profile Estimation and Method Comparison

Reference composition and reference‐to‐mixture compatibility are known to alter deconvolution performance [29]. For profile nonnegative least squares (NNLS), a single CD45‐derived reference was used for both preparations. Mean linear‐scale expression profiles were constructed for all 39 annotated discovery states using the union of their fixed markers (352 informative genes shared with bulk data), and each profile was normalized to unit column sum. Bulk expression over the same genes was also normalized before decomposition with pracma 2.4.6. Nonnegative coefficients were renormalized over all 39 reference states; the 13 eligible states were then evaluated. These relative transcript‐mixture coefficients and the all‐captured‐cell fractions have different denominators and were compared by Spearman rank correlation, not treated as interchangeable cell percentages. For the broader preparation, coefficients and observed fractions were averaged separately within patient before comparison.

ProM was evaluated separately with its source implementation [30]. State‐specific mean profiles and between‐sample variances were derived from the CD45‐enriched object; when a state variance was not estimable, the corresponding parent‐lineage variance was used, and any remaining nonfinite values were set to zero. ProM was run in profile‐only and combined modes with bulk variance enabled, no additional transformation, 20 markers, and the simulation marker option; fitdistrplus 1.2‐6 supported the distribution fitting. CD45‐enriched and cross‐preparation pairs used the same reference basis and predefined markers. Module scores, profile‐NNLS, ProM profile‐only, and ProM combined were compared by state, preparation, estimability, rank, and correlation with matched fractions.

### 2.10. Clinical Association and Survival Analysis in GSE176307

The 13 state scores and the parent‐lineage scores were standardized before clinical modeling. Total immune expression was defined as the mean standardized expression of PTPRC, LAPTM5, LST1, TYROBP, CD3D, CD3E, CD79A, and NKG7. Objective response was complete or partial response versus nonresponse. Logistic regression estimated the response odds ratio for a one‐standard‐deviation increase in each state score. Models were fitted without adjustment, with total immune expression, and, for nonsingleton subtypes, with both total immune and parent‐lineage scores. Base and expanded models were fitted on identical complete‐case sets; the difference in apparent AUC was used descriptively rather than as external validation.

Overall and progression‐free survival were analyzed with continuous standardized scores using survival 3.8‐6. Univariable Cox models, without clinical or immune covariate adjustment, returned hazard ratios per standard‐deviation increase and Wald 95% confidence intervals. Proportional hazards were assessed with scaled Schoenfeld residuals. Predictor dependence was quantified by pairwise Spearman correlation; a clinical model was flagged as unstable when the maximum absolute correlation among its predictors was at least 0.90.

### 2.11. Incremental Discrimination of CR Versus Progression in IMvigor210

The IMvigor210 CR‐versus‐PD analysis used the same standardized state, parent‐lineage, and total‐immune scores. Logistic models were fitted without adjustment, with total immune expression, with total immune plus parent lineage, and with total immune, parent lineage, and tumor mutational burden when applicable. All comparisons between nested models used the same complete‐case subset. Tumor mutational burden was standardized, and models containing it were restricted to the 150 patients with available values. Odds ratios, Wald intervals, *p* values, apparent AUCs, and changes in AUC were reported for the prespecified states.

The added predictive value of Plasma was tested by 20 repeats of stratified five‐fold cross‐validation with seed 20260715. Four models were compared: total immune expression, total immune plus Plasma, total immune plus tumor mutational burden, and all three predictors. Model definitions were held fixed during resampling to limit selection‐induced cross‐validation bias [31]. Within each analysis population, the nested models shared identical folds. Gene‐wise scaling and tumor‐mutational‐burden standardization used training‐fold estimates only. Each patient received one held‐out probability per repeat; these 20 probabilities were averaged, and all reported metrics and curves were calculated from the resulting single probability per patient, not from averages of fold‐level metrics. ROC AUC was calculated with pROC 1.19.0.1 [32], and average precision was included because CRs were uncommon. Brier score and observed response across predicted‐probability quintiles assessed prediction error and calibration [33]. Interpretation followed the distinction between model development and validation emphasized by TRIPOD [34] and the risk domains described in PROBAST [35].

### 2.12. Secondary Clinical Analyses and Evidence Synthesis

The secondary analyses used separate biologically specified gene sets and composite contrasts, not the 13 CD45‐derived state‐marker modules. They examined antigen presentation, effector immunity, and related programs in other compartments and treatment settings. In GSE176178, baseline BCG response was evaluated with Mann–Whitney tests and AUC, with molecular‐subtype effects considered in light of prior BCG‐response work [36]. GSE244895 and GSE199471 were analyzed with paired Wilcoxon tests for within‐patient longitudinal change. GSE145281 used response‐group comparisons and AUCs for peripheral‐blood programs and cell fractions. GSE269877 was used descriptively to record cell‐type‐specific expression and program coverage because its structure did not support a directly comparable clinical test. *p* values were adjusted within each cohort‐analysis family. Unmeasured combinations were recorded as unavailable. The summary matrix integrated donor support, fixed‐module and nested transfer, scoring robustness, parent‐lineage specificity, cross‐preparation results, profile‐NNLS, ProM, and adjusted clinical associations. Results were classified as supported, unsupported, uncertain, not applicable, or not estimable using the study criteria; unsupported denoted failure to meet those criteria rather than evidence that an association was absent.

### 2.13. Two‐Stage Prioritization of an Experimental Candidate

The experimental‐candidate screen began with TCGA‐BLCA primary tumors and adjacent normal tissues. Differential expression was estimated with limma 3.64.3 [37] using empirical‐Bayes variance moderation [38]. The initial screen excluded clinical outcomes and required six filters: tumor log2 fold change of at least 0.50, differential‐expression false‐discovery rate below 0.05, median paired tumor‐minus‐normal difference above 0.25, paired Wilcoxon *p* below 0.10, detection in at least 20% of epithelial cells, and mean epithelial log‐normalized expression of at least 0.15. The 429 genes meeting these criteria formed the candidate pool for the subsequent outcome‐informed assessment.

Candidates were subsequently compared across seven evidence domains: TCGA tumor upregulation, epithelial compatibility, independent upregulation in GSE13507, adjusted adverse association with TCGA overall survival, adjusted adverse association with TCGA progression‐free interval, adverse continuous overall‐survival direction in GSE13507, and association with the immune programs measured in GSE176307. TCGA survival models adjusted for age, sex, and ordinal pathologic stage; GSE13507 models additionally considered invasiveness and grade when complete. P4HB and LY6E met all seven criteria. P4HB was not pursued because its prognostic and functional role in bladder cancer had already been reported [39], and RPN2 was not advanced because published bladder cancer growth and metastasis experiments overlapped the proposed assays [40].

### 2.14. LY6E Expression, Survival, and Immune‐Context Analyses

LY6E expression was compared between 407 TCGA primary tumors and 19 adjacent normal tissues and, separately, within the 19 patient‐matched pairs. GSE13507 provided an external comparison among 165 primary tumors, 58 surrounding normal mucosae, and nine benign bladder controls after exclusion of GSM340542 because its deposited tissue, organism, and assay annotations were internally inconsistent and did not establish bladder origin. Where several array probes mapped to one gene, the probe with the largest interquartile range was retained; ties used the first probe in source order. In the 29,452‐cell cross‐preparation object, LY6E mean expression and detection frequency were summarized across 15 deposited lineages and separately within epithelial and immune compartments.

Continuous Cox models were fitted for TCGA overall survival and progression‐free interval, GSE13507 overall survival, and GSE176307 overall and progression‐free survival. A GSE13507 Kaplan–Meier curve used the cohort median as the fixed expression cut point. Response associations were estimated in GSE176307 before and after immune‐context adjustment and in IMvigor210 before and after tumor‐mutational‐burden adjustment. The different endpoints—objective response in GSE176307 and CR versus PD in the retained IMvigor210 subset—were kept distinct when interpreting these associations.

### 2.15. Cell Culture

Human bladder cancer cell lines T24 (ATCC HTB‐4), 5637 (ATCC HTB‐9), J82 (ATCC HTB‐1), and UM‐UC‐3 (ATCC CRL‐1749) were used. T24 cells were maintained in McCoy′s 5A Modified Medium (Gibco, 16600082), 5637 cells in RPMI 1640 (Gibco, 11875093), and J82 and UM‐UC‐3 cells in Minimum Essential Medium (Gibco, 11095080). Each basal medium contained 10% fetal bovine serum (Gibco, 10099141) and 1% penicillin‐streptomycin (Gibco, 15140122). Cells were cultured at 37°C in humidified 5% carbon dioxide, with medium renewed every 2–3 days. Cultures at approximately 80%–90% confluence were washed with phosphate‐buffered saline (Gibco, 10010023), detached with 0.25% trypsin‐EDTA (Gibco, 25200056), and subcultured. Cell‐line identity was confirmed by short tandem repeat profiling, and mycoplasma was monitored with the MycoAlert PLUS Mycoplasma Detection Kit (Lonza, LT07‐710). Only cultures confirmed to be mycoplasma negative were used.

### 2.16. Transient LY6E knockdown and rescue

Five thousand, six hundred and thirty seven and J82 cells were seeded 24 h before transfection and transfected at approximately 60%–70% confluence. GenePharma supplied plasmids encoding two nonoverlapping short hairpin RNAs against human LY6E (sh‐LY6E‐1 and sh‐LY6E‐2) and a nontargeting control (sh‐NC). For knockdown, cells in six‐well plates received 2 *μ*g of plasmid per well with Lipofectamine 3000 (Invitrogen, L3000015) in Opti‐MEM I Reduced Serum Medium (Gibco, 31985070). The transfection mixture was replaced by complete medium after 6 h, and LY6E transcript abundance was measured 48 h after transfection. Both shRNAs were used for knockdown validation and the initial functional assays.

For rescue, full‐length human LY6E coding sequence was cloned into pcDNA3.1(+) (Invitrogen, V79020). Synonymous substitutions were introduced into the sh‐LY6E‐1 recognition region without changing the encoded protein sequence, producing an shRNA‐resistant construct; empty pcDNA3.1(+) served as the vector control. Cells were cotransfected with 1 *μ*g of sh‐NC or sh‐LY6E‐1 and 1 *μ*g of empty vector or LY6E‐rescue plasmid per well. The three groups were sh‐NC + Vector, sh‐LY6E‐1 + Vector, and sh‐LY6E‐1 + LY6E‐rescue. Restoration of LY6E transcript abundance was assessed 48 h after transfection before cells were reseeded for colony formation.

### 2.17. Reverse Transcription Quantitative PCR

Total RNA was isolated with RNAiso Plus (Takara Bio, 9109), and concentration and purity were measured with a NanoDrop One spectrophotometer. One microgram (1 *μ*g) of RNA was treated for genomic‐DNA removal and reverse transcribed with the PrimeScript RT Reagent Kit with gDNA Eraser (Takara Bio, RR047A): 42°C for 2 min, 37°C for 15 min, and 85°C for 5 s. Quantitative PCR was performed on a QuantStudio 5 system with TB Green Premix Ex Taq II (Takara Bio, RR820A). Each 20‐*μ*L reaction contained 10 *μ*L of 2× premix, 0.8 *μ*L each of forward and reverse primer, 2 *μ*L of diluted cDNA, and 6.4 *μ*L of nuclease‐free water. Cycling comprised 95°C for 30 s, followed by 40 cycles of 95°C for 5 s and 60°C for 30 s, and then melt‐curve analysis.

Primers were synthesized by Sangon Biotech, and all sequences are written 5 ^′^‐3 ^′^: LY6E forward, CAGGCTGCTTTGGTTTGTGACCTC; LY6E reverse, AAGCAGGAGAAGCACATCAGCGAG; GAPDH forward, GTCTCCTCTGACTTCAACAGCG; and GAPDH reverse, ACCACCCTGTTGCTGTAGCCAA. Relative LY6E abundance was normalized to GAPDH by the 2^−*ΔΔ*Ct method. Three independent biological experiments were performed, with each sample measured in technical triplicate.

### 2.18. CCK‐8 Assay

At 24 h after knockdown transfection, 5637 and J82 cells were counted and seeded into 96‐well plates at 2 × 10∧3 cells per well in 100 *μ*L of complete medium, with five replicate wells per condition. At 24, 48, 72, and 96 h after seeding, 10 *μ*L of Cell Counting Kit‐8 reagent (Beyotime Biotechnology, C0038) was added to each well. Plates were incubated for 1.5 h at 37°C protected from light, and absorbance at 450 nm was recorded with a Multiskan FC microplate photometer (Thermo Fisher Scientific, 51119000). Cell‐free medium containing CCK‐8 reagent was used for background subtraction. Growth curves were generated from corrected absorbance values, and the experiment was independently repeated three times.

### 2.19. Colony‐Formation Assay

At 24 h after the final transfection, single‐cell suspensions were seeded into six‐well plates at 500 cells per well and cultured in complete medium for 10–14 days, with medium renewed every 3 days. Culture was stopped when colonies were clearly visible in control wells without extensive overlap. Cells were washed twice with phosphate‐buffered saline, fixed with 4% paraformaldehyde (Beyotime Biotechnology, P0099) for 15 min, stained with crystal violet (Beyotime Biotechnology, C0121) for 20 min, rinsed, and air‐dried. Whole‐well images were acquired under identical settings. ImageJ was used with a common threshold to measure crystal‐violet‐positive colony area as a percentage of the total well area. Three replicate wells were prepared per condition, and the experiment was independently repeated three times. The same procedure was used for the initial knockdown and rescue experiments.

### 2.20. Transwell Migration Assay

Migration was measured in 24‐well Transwell inserts with 8.0‐*μ*m‐pore polycarbonate membranes (Corning, 3422) without extracellular‐matrix coating. After transfection, 5 × 10∧4 cells in 200 *μ*L of serum‐free medium were placed in the upper chamber, and 600 *μ*L of complete medium containing 10% fetal bovine serum was placed below. After 24 h at 37°C, cells on the upper membrane surface were removed. Migrated cells on the lower surface were fixed with 4% paraformaldehyde for 15 min and stained with crystal violet for 20 min. Five nonoverlapping fields were selected at random from each membrane under identical magnification and acquisition settings. Crystal‐violet‐positive migrated area was quantified with ImageJ. Three replicate inserts were included per condition, and the experiment was independently repeated three times.

### 2.21. Annexin V‐FITC/Propidium‐Iodide Apoptosis Assay

Apoptosis was assessed 48 h after transfection with the Annexin V‐FITC Apoptosis Detection Kit (Beyotime Biotechnology, C1062L). Floating cells and adherent 5637 or J82 cells were collected together; adherent cells were detached with 0.25% trypsin without EDTA (Gibco, 15050065). After two washes with pre‐cooled phosphate‐buffered saline, 1 × 10∧5 to 5 × 10∧5 cells were resuspended in 195 *μ*L of binding buffer, mixed with 5 *μ*L of Annexin V‐FITC and 10 *μ*L of propidium iodide, and incubated for 15 min at room temperature in the dark. Samples were analyzed within 1 h on a FACSCanto II flow cytometer. Unstained and single‐stained controls were used for compensation and quadrant placement, and at least 10,000 singlet‐cell events were acquired per sample. FlowJo 10.8.1 was used for analysis. Annexin V‐FITC‐negative/propidium‐iodide‐negative cells were classified as viable, Annexin V‐FITC‐positive/propidium‐iodide‐negative cells as early apoptotic, and double‐positive cells as late apoptotic; total apoptosis was the sum of early and late fractions. The experiment was independently repeated three times.

### 2.22. Statistical Analysis

All statistical tests were two‐sided. Spearman correlation was used for matched transfer analyses because sample sizes were small and the score scales differed. Between‐group cohort comparisons used the Mann–Whitney or Wilcoxon rank‐sum test, paired comparisons used the Wilcoxon signed‐rank test, response models used logistic regression, and time‐to‐event models used Cox proportional‐hazards regression; logistic and Cox estimates are reported with Wald 95% confidence intervals. Exact permutation inference was used for the five‐patient cross‐preparation analysis, and bootstrap intervals followed the resampling schemes described above. Benjamini–Hochberg correction included all 13 eligible states separately for each GSE176307 survival endpoint and each cohort‐specific response‐model tier, not only the four states passing the technical screen. Secondary tests were corrected within their cohort‐analysis families; biologically distinct cohorts and endpoints were not combined. Missing covariates were handled by complete‐case analysis, and no values were imputed. Cell‐assay data are presented as mean ± standard deviation across three independent biological experiments, with the experiment—not an individual qPCR reaction, well, insert, microscopic field, or cytometry event—as the unit of analysis. Two‐group comparisons used an unpaired two‐tailed Student *t*‐test. Relative LY6E expression, colony‐positive area, migrated area, and apoptotic fraction across three or more groups were evaluated by one‐way analysis of variance followed by Tukey multiple comparisons within each cell line; CCK‐8 absorbance across treatment groups and time points was evaluated by two‐way analysis of variance followed by Tukey multiple comparisons. Cell‐assay significance was defined as *p* < 0.05. Analyses were performed in R 4.5.1 and GraphPad Prism 9.5.0. Random procedures used fixed seeds recorded in the scripts. Figures were generated with ggplot2 4.0.2 and assembled with patchwork 1.3.2; rasterization of large point layers affected display only. Pair maps, marker lists, source tables, software versions, and session information were retained for each analysis.

## 3. Results

### 3.1. The Multicohort Design Separated Technical Transfer From Clinical Evaluation

GSE223971 was the only resource with exact single‐cell/bulk pairs, whereas response, survival, longitudinal sampling, and tumor mutational burden were distributed across the other cohorts (Figure 1A,B). The CD45‐enriched and broader GSE223971 preparations contained 43,827 and 29,452 cells, respectively, but the paired analyses were based on eight independent CD45‐enriched pairs and six broader preparation samples from five patients. The two preparations also differed in their representation of immune, epithelial, and stromal compartments (Figure 1C). Clinical class imbalance was evident in both treatment analyses: GSE176307 contained 16 objective responders among 89 patients, and the IMvigor210 subset contained 25 CR and 167 PD cases (Figure 1D). Exact pairing was available only for the technical benchmark, not for the clinical cohorts (Figure 1B).

**Figure 1:**
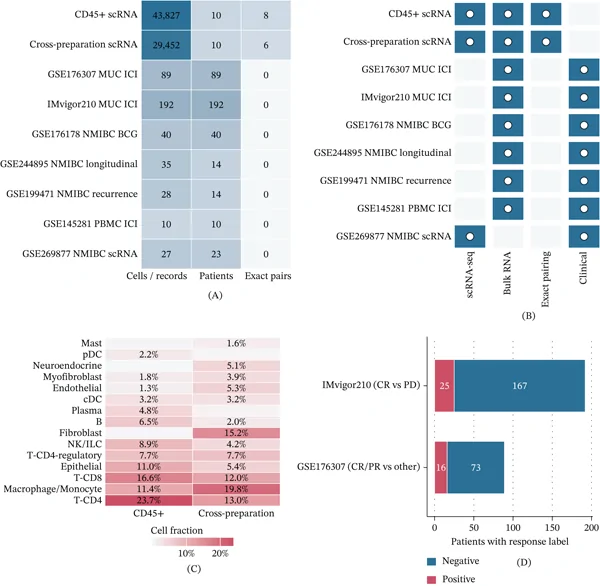
Multicohort design for technical transfer and clinical evaluation. (A) Numbers of measured cells or records, independent patients, and exact single‐cell/bulk pairs. (B) Availability of single‐cell RNA sequencing, bulk RNA, exact pairing, and clinical data. (C) Major‐lineage composition of the CD45‐enriched and broader GSE223971 preparations. (D) Objective‐response counts in GSE176307 and CR‐versus‐PD counts in the selected IMvigor210 subset. The cross‐preparation analysis comprised six samples from five independent patients.

### 3.2. Donor‐Aware Quality Review Retained 13 States for the Transfer Benchmark

The CD45‐enriched atlas resolved lymphoid, myeloid, dendritic, stromal, and Plasma compartments (Figure 2A). Eligible minor states were distributed across the UMAP rather than concentrated in one region (Figure 2B). The marker dot plot showed distinct expression patterns for the states that entered module derivation (Figure 2C). Sample‐level composition varied substantially across specimens (Figure 2D).

**Figure 2:**
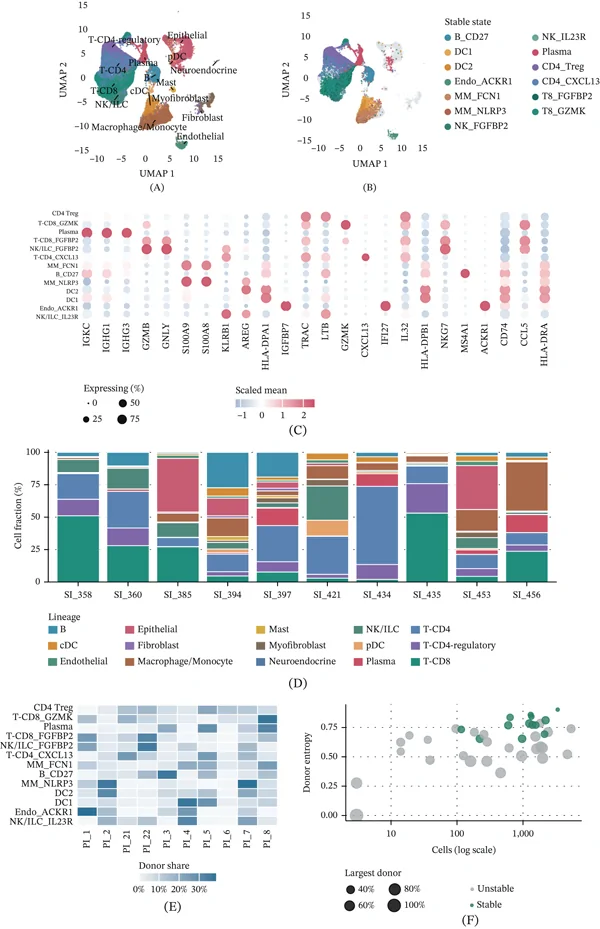
Donor‐aware quality review defines the 13‐state CD45‐enriched benchmark. (A, B) Deposited UMAP coordinates colored by major lineage and eligible minor state, respectively. (C) Marker‐expression dot plot for the eligible states; dot size indicates the percentage of cells with detectable expression and color indicates scaled mean expression. (D) Major‐lineage composition by sample. (E) Patient contribution to each annotated minor state. (F) State‐level cell count, normalized donor entropy, and maximum donor share. UMAP was used for display only; no additional clustering or UMAP‐based filtering was performed.

The donor‐contribution matrix identified states supported across patients and states dominated by a smaller subset of donors (Figure 2E). Cell count, donor entropy, and maximum donor share provided complementary views of that structure (Figure 2F). Applying the minimum cell count, minimum patient count, and maximum donor‐share rules retained 13 of 39 states. These 13 states, rather than all deposited annotations, comprised the predefined transfer set.

### 3.3. The Broader Preparation Altered Both Cellular Composition and State Detectability

The second GSE223971 object contained a larger stromal component and altered epithelial representation than the CD45‐enriched atlas (Figure 3A). Only a subset of the CD45‐derived immune states was recoverable in this broader mixture (Figure 3B). Sample‐level composition confirmed that the added compartments were unevenly represented rather than introduced at a constant proportion (Figure 3C). Median lineage fractions quantified this compositional shift (Figure 3D). The sample‐to‐patient map covered 13 single‐cell samples from 10 patients, including repeated samples (Figure 3E). State fractions and detection counts showed that several predefined states were absent from individual samples (Figure 3F,G). Only six samples from five patients satisfied the exact single‐cell/bulk matching rule (Figure 3H), requiring patient aggregation and exact patient‐level inference in subsequent analyses.

**Figure 3:**
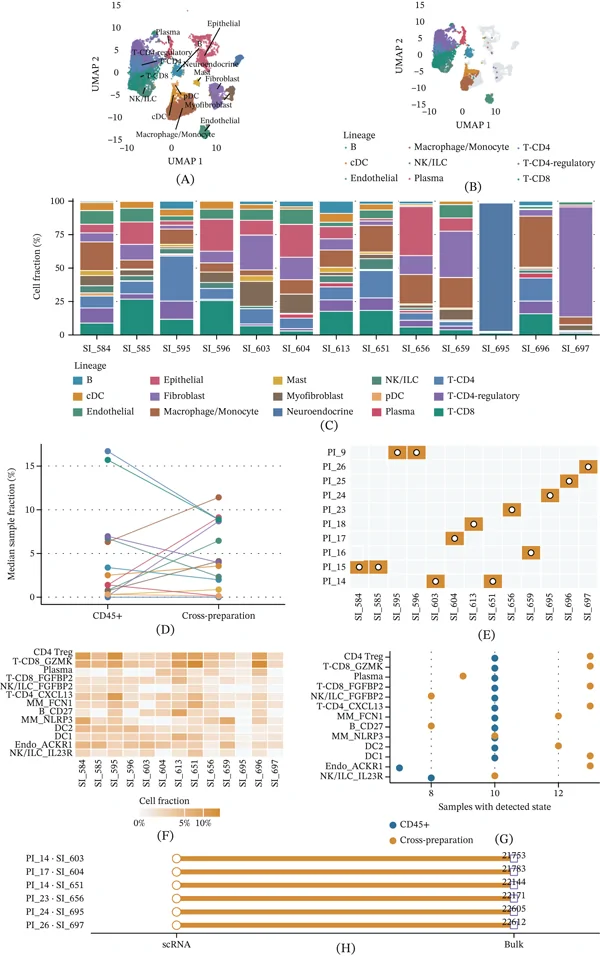
The broader GSE223971 preparation changes cellular composition and state coverage. (A, B) UMAPs colored by major lineage and coverage of the CD45‐derived eligible states. (C) Sample‐level lineage composition. (D) Median lineage fractions in the two preparations. (E) Mapping of all 13 broader preparation single‐cell samples to 10 patients; the six exact bulk matches are identified in H. (F) All‐captured‐cell fractions of the predefined states across the 13 broader preparation samples. (G) Number of samples with each state detected in the two preparations. (H) Exact linkage between the single‐cell samples and bulk RNA profiles. Differences between preparations are descriptive because enrichment and tissue composition changed simultaneously.

### 3.4. Parent‐Lineage Adjustment Did Not Support Subtype‐Specific Recovery

Correlations with all‐captured‐cell fractions were frequently stronger than correlations with within‐parent fractions (Figure 4A). Subtracting the parent‐lineage score or residualizing the state score against it did not consistently restore within‐parent agreement (Figure 4B). The matched DC2 and DC1 examples showed limited concordance between bulk scores and all‐captured‐cell fractions (Figure 4C,D). Their contributions to the captured‐cell pool varied across patients (Figure 4E).

**Figure 4:**
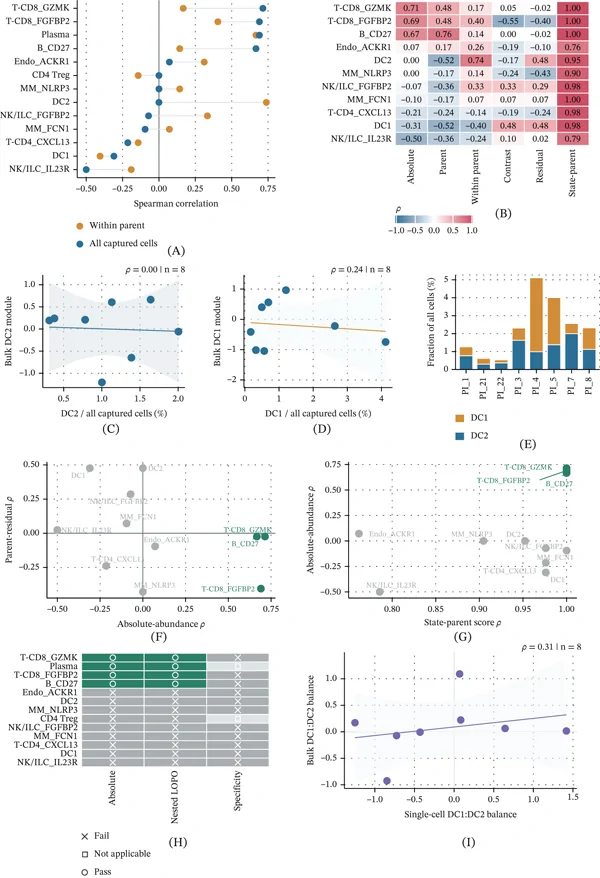
Parent‐lineage adjustment does not support subtype‐specific recovery. (A) State‐wise Spearman correlations of bulk scores with all‐captured‐cell and within‐parent fractions. (B) Correlations with within‐parent fractions after subtracting the parent‐lineage score or residualizing the state score against it. (C, D) DC2 and DC1 all‐captured‐cell fractions plotted against their matched bulk scores. (E) DC1 and DC2 fractions among all captured cells in each matched patient. (F) Absolute‐abundance versus parent‐residualized correlations for the 11 testable subtypes, highlighting the three subtypes retained by the initial transfer screen. (G) Coupling between state and parent‐lineage scores. (H) Combined fixed‐transfer, nested‐transfer, and specificity decisions. (I) Recovery of the DC1: DC2 balance. Dashed lines denote exploratory screening thresholds. Singleton lineages, including Plasma, were classified as not applicable for within‐parent specificity.

The three evaluable subtypes retained by the initial abundance screen fell below the specificity threshold; Plasma was not evaluable as a singleton lineage (Figure 4F). T‐CD8_GZMK, T‐CD8_FGFBP2, and B_CD27 were perfectly rank‐correlated with their parent‐lineage scores in the eight pairs (rho = 1.000 for each; Figure 4G). Pair‐level views therefore supported broad lineage‐associated ordering but not independent recovery of the intended subtype (Figure 4G). When transfer, nested rederivation, and specificity were considered together, none of the 11 nonsingleton subtypes met the specificity criteria (Figure 4H). The DC1: DC2 balance was likewise not reproduced reliably in bulk RNA (Figure 4I).

### 3.5. Four State Modules Met the Initial Transfer Criteria Despite Wide Uncertainty

Across the 13 eligible states, the strongest 20‐gene z‐mean correlations were observed for T‐CD8_GZMK (rho = 0.714), Plasma (rho = 0.690), T‐CD8_FGFBP2 (rho = 0.690), and B_CD27 (rho = 0.667) (Figure 5A). These four met the predefined screening criteria (Figure 5A), but the lower limits of their 95% bootstrap intervals were −0.105, −0.037, −0.151, and −0.215, respectively. Thus, every inferential interval included zero despite positive lower bounds for the 80% screening interval. The direction of transfer nevertheless remained consistent under z‐mean, rank‐mean, and mean‐log scoring (Figure 5B). Pair‐level rank error varied by state and patient, without one specimen explaining all four signals (Figure 5C). Leave‐one‐pair‐out minima ranged from 0.500 to 0.571 (Figure 5D), whereas state size and donor support showed no simple relationship with transfer performance (Figure 5E). The matched plots for Plasma, T‐CD8_GZMK, T‐CD8_FGFBP2, and B_CD27 showed monotonic but imperfect ordering across the eight patients (Figure 5F, G, H, I).

**Figure 5:**
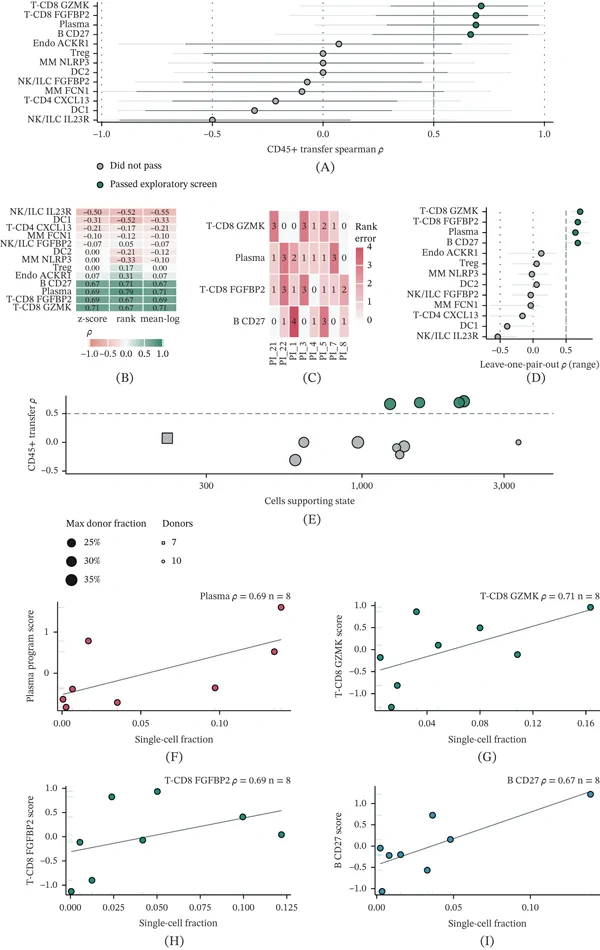
Matched transfer estimates for the eligible state modules. (A) Correlations with pair‐bootstrap 80% screening intervals and 95% inferential intervals. (B) State‐wise correlations obtained with z‐mean, rank‐mean, and mean‐log scoring. (C) Pair‐level rank error for T‐CD8_GZMK, Plasma, T‐CD8_FGFBP2, and B_CD27. (D) Correlation ranges after leaving out each pair in turn. (E) Transfer correlation in relation to state size and donor support. (F, G, H, I) Matched single‐cell fractions and bulk scores for Plasma, T‐CD8_GZMK, T‐CD8_FGFBP2, and B_CD27, respectively. Bootstrap intervals were calculated from 5000 pair‐level resamples. The lower screening bound was positive for all four states, whereas every 95% interval included zero.

### 3.6. Marker Membership Remained Stable During Nested Rederivation

Module‐length analysis showed that small signatures were more sensitive to the number of retained genes and to the scoring rule (Figure 6A). After excluding the held‐out patient from marker derivation and bulk standardization, nested correlations remained positive for T‐CD8_GZMK (rho = 0.571), Plasma (rho = 0.690), T‐CD8_FGFBP2 (rho = 0.690), and B_CD27 (rho = 0.643) (Figure 6B). Plasma had the highest median Jaccard overlap with its full‐data marker set (1.000), followed by T‐CD8_FGFBP2 (0.905), B_CD27 (0.861), and T‐CD8_GZMK (0.779) (Figure 6C). Gene‐level recurrence across folds produced the same overall ranking of stability (Figure 6D). With 20 markers, all four states remained supported under the three scoring rules (Figure 6A), and their held‐out predictions remained positively ordered (Figure 6E, F, G, H).

**Figure 6:**
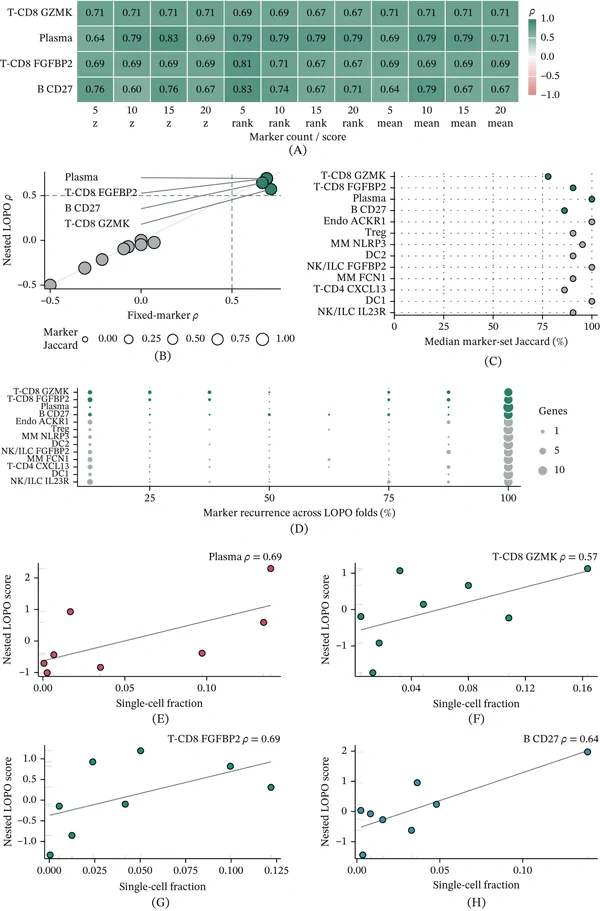
Marker stability and held‐out scores during leave‐one‐patient‐out rederivation. (A) Sensitivity of transfer correlations to module length and scoring rule. (B) Fixed‐module correlation compared with the nested held‐out correlation. (C) Jaccard overlap between each fold‐specific module and the corresponding full‐data module. (D) Gene‐level recurrence across the nested folds; dot area represents the number of genes at each recurrence frequency. (E, F, G, H) Held‐out predictions for Plasma, T‐CD8_GZMK, T‐CD8_FGFBP2, and B_CD27, respectively. Each fold excluded the held‐out patient from marker derivation, leaving nine single‐cell donors; score standardization used only the other seven paired bulk samples.

### 3.7. Plasma Was the Only Module Retained After the Cellular Mixture Changed

Transfer performance in the CD45‐enriched pairs did not reliably predict performance in the broader preparation (Figure 7A). Sample‐level correlations were positive for several states, including Plasma (rho = 0.714) and B_CD27 (rho = 0.543) (Figure 7B). Clustered‐bootstrap intervals were wide (Figure 7B), and estimates changed after duplicate samples from the same patient were aggregated (Figure 7C).

**Figure 7:**
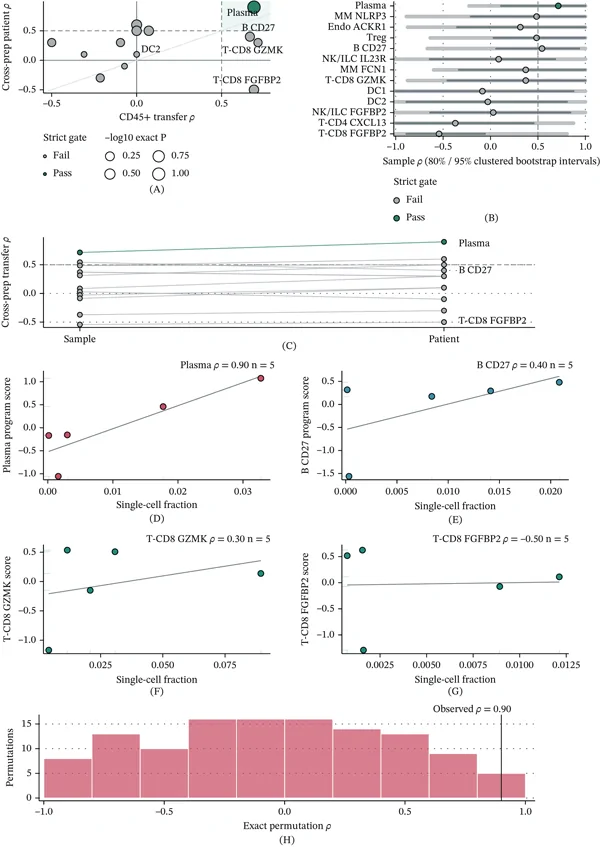
Plasma is the only module retained after transfer to a different cellular mixture. (A) State‐wise comparison of CD45‐enriched and cross‐preparation correlations. (B) Patient‐clustered bootstrap intervals for the sample‐level estimates. (C) Change in correlation after repeated samples were aggregated by patient. (D, E, F, G) Patient‐level matched plots for Plasma, B_CD27, T‐CD8_GZMK, and T‐CD8_FGFBP2. (H) Exact permutation distribution for the Plasma patient‐level correlation across all 120 labelings. Five patients were the independent units; the exact two‐sided Plasma *p* value was 0.083.

At the patient level, Plasma reached rho = 0.900, whereas B_CD27, T‐CD8_GZMK, and T‐CD8_FGFBP2 reached 0.400, 0.300, and −0.500, respectively (Figure 7D, E, F, G). Across all 120 patient‐label permutations, the observed Plasma correlation gave an exact two‐sided *p* value of 0.083 (Figure 7H). Plasma was the only module to meet the patient‐level exploratory transfer criteria.

### 3.8. Transfer Estimates Remained Sensitive to the Scoring and Reference Method

No method ranked uniformly first across states and preparations (Figure 8A). State‐wise ranges widened when module scores were compared with profile‐based estimates, and method ranks changed between the CD45‐enriched and broader mixtures (Figure 8A). ProM combined estimated Plasma with rho = 0.786 in the CD45‐enriched pairs (Figure 8B) and rho = 0.700 after patient aggregation in the cross‐preparation set, where the exact *p* value was 0.233 (Figure 8C). Profile‐only and combined ProM were concordant for some states but diverged for others (Figure 8D). With the fixed CD45 reference, Plasma profile‐NNLS correlations were 0.627 in the eight discovery pairs and 0.975 across the five broader preparation patients. Plasma was the only state for which the z‐mean module and profile‐NNLS were positively concordant in both technical settings (Figure 8E). The number of estimable states differed by method (Figure 8F).

**Figure 8:**
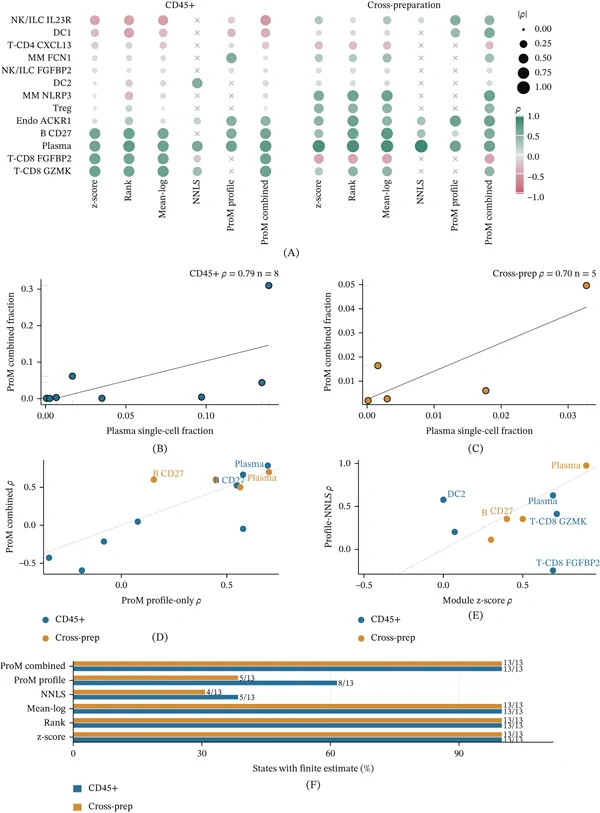
Transfer estimates vary across module scores and reference‐profile methods. (A) State‐by‐method correlations in the CD45‐enriched and cross‐preparation settings. (B, C) Matched Plasma estimates from ProM combined in the CD45‐enriched and cross‐preparation datasets. (D) Comparison of ProM profile‐only and combined modes. (E) Z‐mean module correlation compared with profile‐NNLS correlation. (F) Proportion of eligible states estimable by each approach. Cross‐preparation method comparisons use five patient‐level observations. NNLS uses the same 39‐state CD45 reference in both preparations. The displayed comparison includes only the three module scores, profile‐NNLS, and the two implemented ProM modes.

### 3.9. GSE176307 Separated Prognostic Immune Context From Treatment‐Response Association

None of the four initially transferred states showed a statistically supported association with objective response in GSE176307. Unadjusted odds ratios per standard deviation were 1.242 for T‐CD8_GZMK, 1.146 for Plasma, 1.261 for T‐CD8_FGFBP2, and 1.132 for B_CD27, with all *p* values above 0.40 (Figure 9A). Adjustment for total immune expression and parent lineage did not produce stable independent effects (Figure 9A), and Plasma distributions overlapped extensively between response groups (Figure 9B).

**Figure 9:**
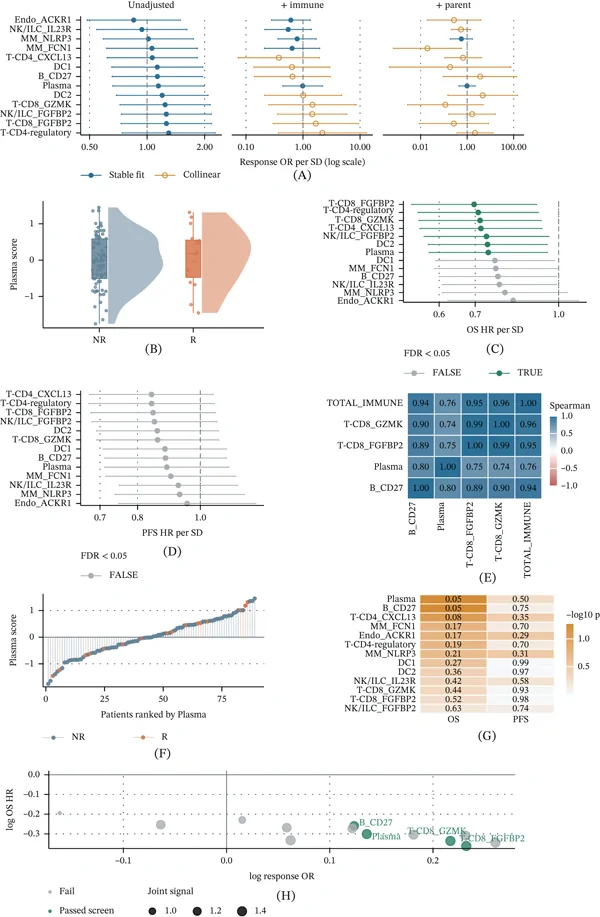
GSE176307 distinguishes survival‐associated immune context from objective‐response prediction. (A) Unadjusted and adjusted objective‐response odds ratios per standard‐deviation increase in each state score. (B) Plasma‐score distribution in responders and nonresponders. (C, D) Unadjusted continuous hazard ratios for overall and progression‐free survival. (E) Spearman correlations among the four screened state scores and the total immune score. (F) Plasma scores across patients ranked from low to high, with objective‐response annotations. (G) Schoenfeld‐residual‐based proportional‐hazards test *p* values for overall and progression‐free survival. (H) Joint display of response and overall‐survival effects. The cohort contained 89 patients, 16 responders, 57 overall‐survival events, and 78 progression‐free‐survival events among 88 evaluable patients.

The survival results differed from the response results. Unadjusted overall‐survival hazard ratios were 0.715 for T‐CD8_GZMK (95% CI 0.550–0.928; FDR = 0.040), 0.740 for Plasma (95% CI 0.575–0.952; FDR = 0.042), and 0.697 for T‐CD8_FGFBP2 (95% CI 0.534–0.910; FDR = 0.040); B_CD27 had a similar direction (HR = 0.771, 95% CI 0.600–0.991) but an FDR of 0.053 (Figure 9C). Progression‐free‐survival associations were weaker (Figure 9D). Response‐effect estimates shifted across adjustment tiers (Figure 9A), and the screened scores were correlated with one another and with total immune expression; the Plasma–total immune correlation was rho = 0.757 (Figure 9E). The ranked patient display and proportional‐hazards diagnostics further characterized the response and survival analyses (Figure 9F,G). When the two outcomes were plotted together, a favorable survival direction did not imply response discrimination (Figure 9H).

### 3.10. Plasma Did Not Consistently Improve CR‐Versus‐PD Discrimination in IMvigor210

The unadjusted Plasma odds ratio for CR versus PD was 1.597 (95% CI 1.073–2.376; *p* = 0.021). Adjustment for total immune expression reduced the estimate to 1.286 (95% CI 0.804–2.058; *p* = 0.294; delta AUC = −0.003), and the immune‐plus‐tumor‐mutational‐burden model gave an odds ratio of 1.325 (95% CI 0.684–2.567; *p* = 0.404) (Figure 10A).

**Figure 10:**
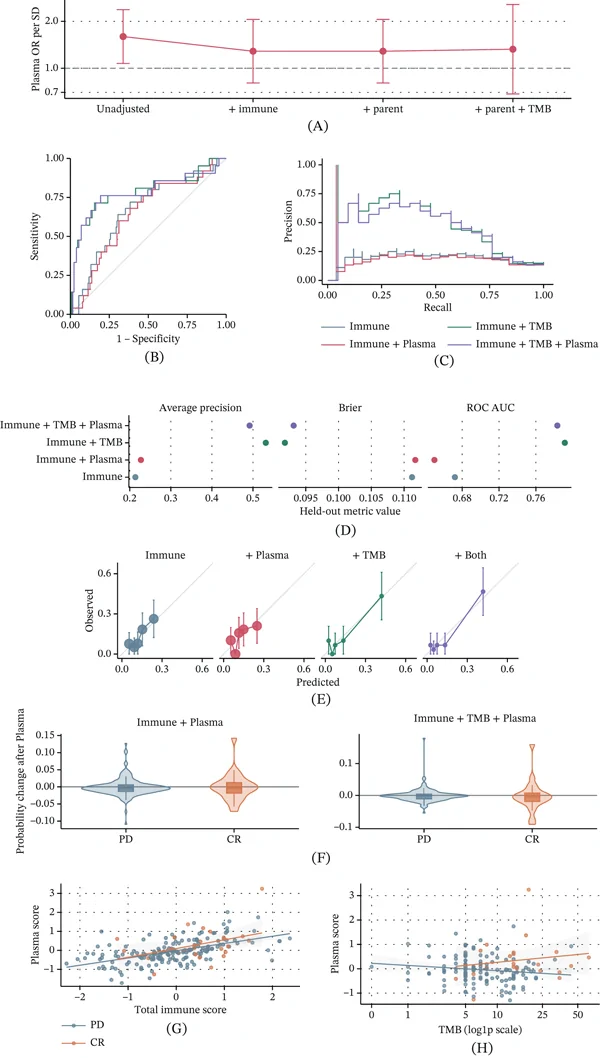
Plasma does not consistently improve CR‐versus‐PD discrimination in IMvigor210. (A) Plasma odds ratios before and after immune and tumor‐mutational‐burden adjustment. (B, C) ROC and precision‐recall curves based on patient‐level averaged held‐out probabilities. (D) Held‐out ROC AUC, average precision, and Brier score displayed on separate scales; larger AUC and average precision and smaller Brier values indicate better performance. (E) Observed CR frequency by predicted‐probability quintile. (F) Patient‐level probability change after adding Plasma. (G, H) Plasma in relation to total immune expression and tumor mutational burden; the latter uses a log1p axis to retain zero values. Twenty repeats of stratified five‐fold cross‐validation used shared folds within each analysis population and training‐only scaling. Predictions were averaged across repeats before calculating metrics. The full subset contained 25 CR and 167 PD cases; tumor‐mutational‐burden models used 21 CR and 129 PD cases.

Repeated cross‐validation showed no consistent predictive improvement (Figure 10B,C). Without tumor mutational burden, adding Plasma reduced AUC from 0.672 to 0.650, whereas average precision increased from 0.214 to 0.227; Brier score increased slightly from 0.1112 to 0.1117. Among the 150 complete cases with tumor mutational burden, AUC declined from 0.791 to 0.783 and average precision from 0.531 to 0.492, whereas Brier score increased from 0.0918 to 0.0931 (Figure 10D).

Observed CR frequency across probability quintiles did not show improved calibration (Figure 10E). Adding Plasma produced small, bidirectional patient‐level probability changes (Figure 10F). Plasma correlated with total immune expression (rho = 0.566) but not with tumor mutational burden (rho = −0.042; Figure 10G,H), consistent with a broad immune‐context association rather than independent prediction.

### 3.11. Secondary Clinical Associations Did Not Survive Multiplicity Correction

The cohort‐by‐program matrix contained many unavailable combinations because the datasets measured different biological compartments and endpoints (Figure 11A). Of 35 tests with an available *p* value, only one was nominally below 0.05, and none remained significant after correction within the corresponding analysis family. Baseline BCG‐response AUCs in GSE176178 were all below 0.50 (Figure 11B). Effector_T_NK increased in GSE244895 (median paired change = 0.058; nominal *p* = 0.042), but its corrected *p* value was 0.246 (Figure 11C). No recurrence‐related program change in GSE199471 was significant (Figure 11D). Peripheral‐blood cell fractions were compared separately by response group (Figure 11F). In the 10‐patient peripheral‐blood cohort, Bcell_plasma and APC_effector_minus_IL8 each reached an AUC of 0.88 (Figure 11E), but both had an exact *p* value of 0.056 and a corrected *p* value of 0.306 (Figure 11E). GSE269877 contributed cell‐type expression and gene‐coverage information rather than a comparable module‐level outcome test (Figure 11G).

**Figure 11:**
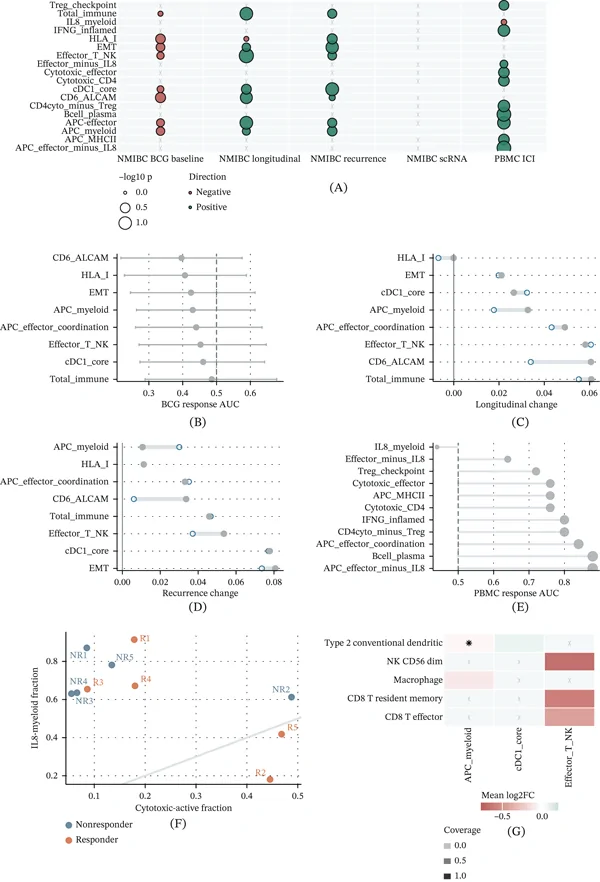
Secondary clinical analyses yield no association after within‐cohort multiplicity control. (A) Direction and nominal significance of predefined program associations across the secondary analyses; crosses denote unavailable tests. (B) Baseline BCG‐response AUCs in GSE176178. (C) Paired program changes in GSE244895. (D) Paired recurrence‐related changes in GSE199471. In C and D, hollow and filled circles show mean and median paired changes, respectively. (E) Peripheral‐blood response AUCs in GSE145281; dot size reflects nominal significance. (F) Selected peripheral‐blood cell fractions by response group. (G) Cell‐type expression and program coverage in GSE269877; crosses mark unavailable combinations, and a star indicates at least one component gene with FDR below 0.05. Gray points in B–E denote FDR of at least 0.05. *p* values were adjusted within each analysis family and were not pooled across biologically distinct cohorts.

### 3.12. Expression and Clinical Evidence Prioritized LY6E for Functional Testing

Initial expression and epithelial filters, which did not include clinical outcomes, reduced the starting set to 429 genes. P4HB and LY6E were the only candidates meeting all seven statistical and biological criteria; the publication‐overlap review then excluded P4HB and retained LY6E (Figure 12A). In TCGA‐BLCA, LY6E was higher in 407 primary tumors than in 19 adjacent normal tissues (log2 fold change = 1.017; FDR = 0.026). The 19 matched pairs showed a median tumor‐minus‐normal difference of 0.629 (*p* = 0.038; Figure 12B). GSE13507 independently showed higher tumor expression across 165 tumors, 58 surrounding normal mucosae, and nine benign controls (tumor − minus − normal median difference = 0.370; *p* = 0.00375; Figure 12C).

**Figure 12:**
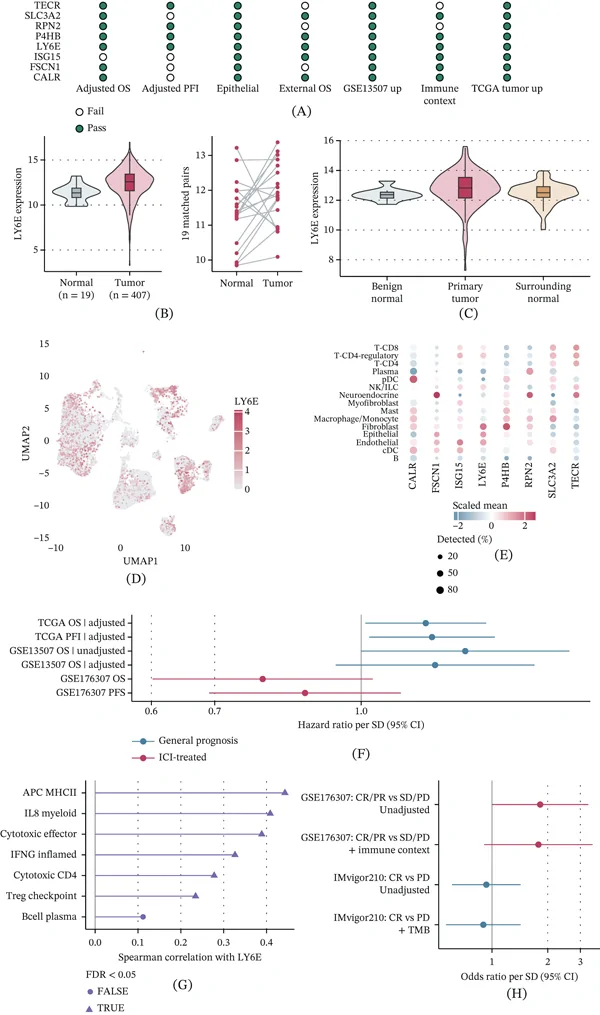
Expression and clinical evidence used to prioritize LY6E. (A) Seven‐domain profile of the leading candidates; filled circles indicate that a criterion was met. (B) LY6E expression in 407 TCGA‐BLCA primary tumors and 19 adjacent normal tissues, with the 19 matched pairs shown separately. (C) Expression in 165 GSE13507 tumors, 58 surrounding normal mucosae, and nine benign bladder controls. Violin plots show the distribution, with box plots indicating the median and interquartile range. (D) LY6E expression on the broader preparation UMAP. (E) Mean expression and detection of candidate genes across 15 annotated lineages. (F) Continuous Cox estimates across TCGA‐BLCA, GSE13507, and GSE176307. (G) Spearman correlations between LY6E and seven immune programs; marker shape indicates FDR below 0.05. (H) Unadjusted and adjusted associations with objective response in GSE176307 and CR versus PD in the retained IMvigor210 subset. Forest plots show effects per standard deviation and 95% confidence intervals. These endpoints were evaluated separately.

LY6E expression occupied both epithelial and immune regions of the cross‐preparation UMAP (Figure 12D). Detection varied across the 15 annotated lineages (Figure 12E); among epithelial cells, 42.9% expressed LY6E and the mean log‐normalized expression was 0.800. Both epithelial and immune populations contributed to the observed LY6E expression pattern.

The GSE13507 median‐split curve was not significant (log − rank *p* = 0.307). Continuous adjusted TCGA models gave hazard ratios per standard deviation of 1.170 for overall survival (95% CI 1.010–1.356; *p* = 0.036) and 1.188 for progression‐free interval (95% CI 1.020–1.385; *p* = 0.027). In GSE13507, the unadjusted overall‐survival hazard ratio was 1.289 (*p* = 0.050), whereas the adjusted estimate was 1.197 (*p* = 0.145); GSE176307 estimates were reversed and nonsignificant (Figure 12F). LY6E correlated with six of seven immune programs, most strongly with APC/MHC‐II (rho = 0.443; Figure 12G). Its GSE176307 response association weakened after immune adjustment, whereas the CR‐versus‐PD association in IMvigor210 had the opposite direction (Figure 12H). Tumor upregulation and epithelial expression supported functional testing of LY6E, although its clinical associations varied across cohorts.

### 3.13. LY6E Knockdown Reduces Growth and Migration and Increases Apoptosis in Bladder Cancer Cells

Among the four bladder cancer cell lines screened, relative LY6E transcript abundance was highest in 5637 (1.987 ± 0.105) and J82 (2.443 ± 0.493) cells, compared with T24 (1.000 ± 0.155) and UM‐UC‐3 (0.647 ± 0.078); 5637 and J82 were therefore taken forward (*n* = 3 independent experiments; Figure 13A). In 5637 cells, sh‐LY6E‐1 and sh‐LY6E‐2 reduced relative expression from 1.000 ± 0.070 to 0.363 ± 0.025 and 0.353 ± 0.038, respectively (both *p* < 0.001; Figure 13B). The corresponding values in J82 were 1.003 ± 0.194, 0.343 ± 0.042, and 0.330 ± 0.078 (both knockdown groups *p* < 0.01; Figure 13C), representing reductions of approximately 64%–67% across the two models.

**Figure 13:**
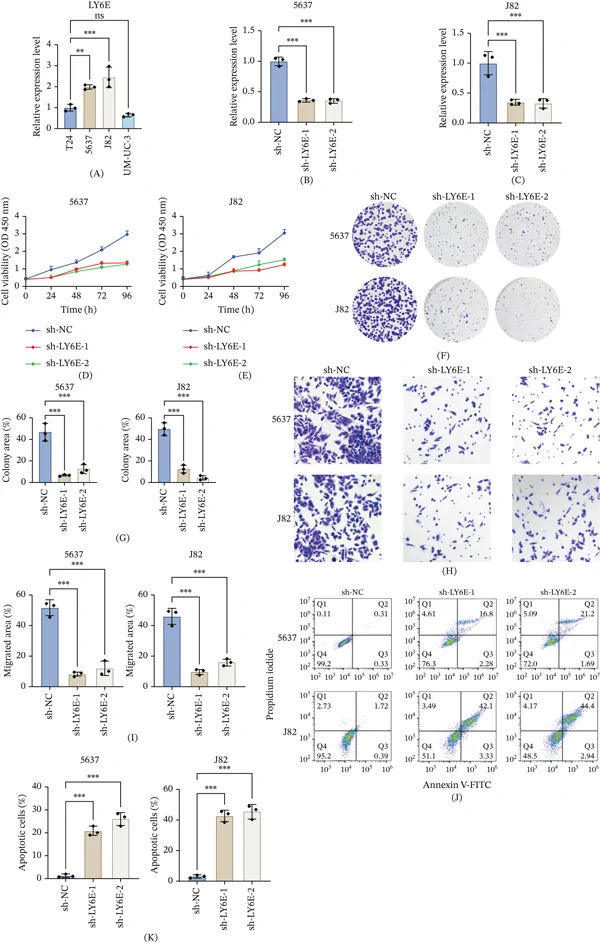
LY6E knockdown reduces growth and migration and increases apoptosis in bladder cancer cells. (A) Relative LY6E transcript abundance in T24, 5637, J82, and UM‐UC‐3 cells. (B, C) RT‐qPCR validation of sh‐LY6E‐1 and sh‐LY6E‐2 in 5637 and J82 cells. (D, E) CCK‐8 absorbance over 96 h after transfection. (F, G) Representative colony‐formation wells and colony‐positive area. (H, I) Representative Transwell fields and migrated area. (J, K) Annexin V‐FITC/propidium‐iodide flow‐cytometry plots and total apoptotic fraction. Data are mean ± SD from three independent biological experiments. One‐way ANOVA with Tukey multiple comparisons was used for multi‐group endpoint comparisons, and CCK‐8 time courses were analyzed by two‐way ANOVA with Tukey multiple comparisons. Double and triple asterisks indicate *p* < 0.01 and *p* < 0.001, respectively; ns, not significant.

Both shRNAs produced progressively lower CCK‐8 absorbance than sh‐NC in 5637 and J82 cells (Figure 13D,E). At 96 h, mean OD450 values were 2.973 ± 0.190 for sh‐NC, 1.363 ± 0.083 for sh‐LY6E‐1, and 1.280 ± 0.046 for sh‐LY6E‐2 in 5637 cells; the corresponding J82 values were 3.057 ± 0.191, 1.260 ± 0.098, and 1.517 ± 0.092. Long‐term clonogenic growth showed the same direction (Figure 13F). Colony‐positive area fell from 46.463*%* ± 8.118*%* in 5637 sh‐NC cells to 6.863*%* ± 0.616*%* and 12.360*%* ± 4.035*%* after sh‐LY6E‐1 and sh‐LY6E‐2, respectively. In J82, it fell from 49.447*%* ± 5.939*%* to 12.493*%* ± 3.552*%* and 4.260*%* ± 1.784*%* (all knockdown comparisons *p* < 0.001; Figure 13G).

LY6E knockdown also reduced Transwell migration in both models (Figure 13H). Migrated area decreased from 51.527*%* ± 4.997*%* in 5637 sh‐NC cells to 8.323*%* ± 1.351*%* and 11.797*%* ± 4.842*%*, and from 46.053*%* ± 5.151*%* in J82 sh‐NC cells to 9.843*%* ± 1.725*%* and 15.917*%* ± 2.226*%* (all *p* < 0.001; Figure 13I). Annexin V/propidium‐iodide flow cytometry showed a reciprocal increase in apoptosis (Figure 13J): The apoptotic fraction rose from 1.287*%* ± 0.677*%* to 20.837*%* ± 2.071*%* and 26.020*%* ± 2.814*%* in 5637 cells and from 3.180*%* ± 0.964*%* to 42.837*%* ± 3.535*%* and 45.617*%* ± 4.774*%* in J82 cells (all *p* < 0.001; Figure 13K). Thus, two independent LY6E‐targeting constructs produced concordant effects on growth, migration, and apoptosis in both tested cell lines.

### 3.14. LY6E Re‐Expression Restores Clonogenic Growth After Knockdown

An shRNA‐resistant LY6E construct was used to test whether the clonogenic phenotype depended on LY6E depletion. In 5637 cells, relative LY6E expression was 1.000 ± 0.184 in sh‐NC + Vector, 0.280 ± 0.066 in sh‐LY6E‐1 + Vector, and 0.973 ± 0.035 in sh‐LY6E‐1 + LY6E‐rescue (*p* < 0.001 for knockdown versus either control or rescue; Figure 14A). The corresponding J82 values were 1.000 ± 0.108, 0.353 ± 0.023, and 0.980 ± 0.180 (*p* < 0.01; Figure 14B).

**Figure 14:**
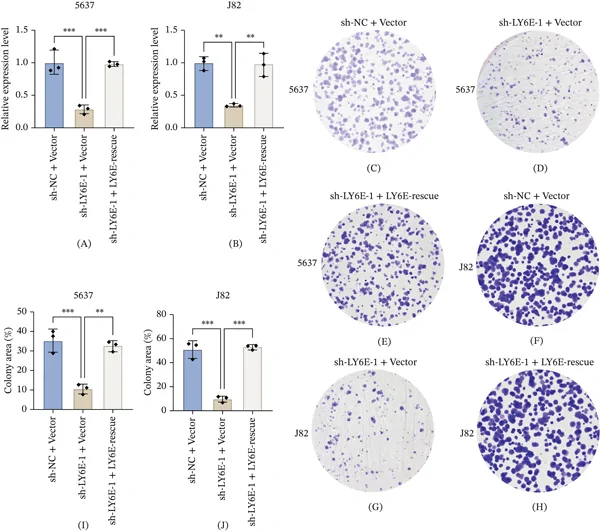
shRNA‐resistant LY6E re‐expression restores clonogenic growth after knockdown. (A, B) Relative LY6E transcript abundance in 5637 and J82 cells transfected with sh‐NC + Vector, sh‐LY6E‐1 + Vector, or sh‐LY6E‐1 + LY6E‐rescue. (C, D, E) Representative colony‐formation wells for the three 5637 groups. (F, G, H) Corresponding J82 wells. (I, J) Colony‐positive area in 5637 and J82 cells. Data are mean ± SD from three independent biological experiments and were analyzed by one‐way ANOVA with Tukey multiple comparisons. Double and triple asterisks indicate *p* < 0.01 and *p* < 0.001, respectively.

Representative colony‐formation images showed loss of clonogenic growth after sh‐LY6E‐1 and recovery after LY6E re‐expression in both 5637 and J82 cells (Figure 14C, D, E, F, G, H). In 5637, colony‐positive area was 35.123*%* ± 6.005*%* in sh‐NC + Vector, 10.540*%* ± 2.446*%* in sh‐LY6E‐1 + Vector, and 32.310*%* ± 2.781*%* after rescue (knockdown versus rescue, *p* < 0.01; Figure 14I). J82 values were 50.737*%* ± 7.278*%*, 9.610*%* ± 2.477*%*, and 52.753*%* ± 2.188*%*, respectively (knockdown versus rescue, *p* < 0.001; Figure 14J). Restoration of transcript abundance and clonogenic growth supports a contribution of LY6E to colony formation in both cell lines.

## 4. Discussion

Matched single‐cell and bulk analyses recovered broad immune‐population signals more consistently than variation within a lineage. Their limited specificity and sensitivity to tissue composition help explain why technical transfer did not translate into consistent gains in response prediction. Four modules were directionally concordant in eight CD45‐enriched pairs, but uncertainty was wide; none of the testable subtypes remained specific after parent‐lineage adjustment, and only Plasma retained a positive module‐level result in the broader preparation. LY6E was evaluated separately: Its depletion produced concordant phenotypes in two bladder cancer cell lines, and an shRNA‐resistant construct restored clonogenic growth.

The parent‐lineage analysis is central to that interpretation. A high matched correlation can arise because a module tracks the size of a broad lineage rather than its internal composition. This was particularly evident for the CD8 and B‐cell modules, whose state scores were nearly indistinguishable from the corresponding parent scores. Simulation studies have shown that cell‐state granularity and reference construction materially affect deconvolution performance [41], and multi‐assay benchmarks have reached a similar conclusion when transcriptomic estimates are compared with orthogonal measurements of cellular abundance [42]. Available methods also encode different assumptions: CIBERSORT uses regression‐based enumeration [43], whereas EPIC explicitly accommodates immune and malignant compartments [44]. MCP‐counter [45] and xCell [46] use marker or enrichment representations, while MuSiC [47], SCDC [48], and CIBERSORTx [49] incorporate single‐cell reference information in different ways. Our empirical comparison was restricted to module scoring, profile‐NNLS, and ProM. Within this set, differences in signal representation altered the estimated agreement with cellular abundance. The close coupling of the CD8 and B‐cell scores to their parent lineages suggests that much of their bulk variation reflected lineage abundance.

Plasma differed from the other transferred states in several respects. Its markers were highly recurrent during leave‐one‐patient‐out rederivation, the positive association persisted across three module‐scoring methods, and profile‐NNLS was concordant in both technical settings. Immunoglobulin‐rich transcription may make this population easier to distinguish from background bulk expression than closely related T‐cell states. The biological meaning of this transcriptional signal also depends on cellular organization. B‐cell‐rich tertiary lymphoid structures have been associated with checkpoint response and survival in melanoma [50], and an independent analysis likewise linked B‐cell organization to treatment response [51]. Such structures contain multiple B‐cell states and interact with T cells, dendritic cells, and stromal compartments [52]. Spatial measurements would be needed to connect the Plasma score to these organized immune structures.

The broader preparation analysis examined robustness to a changed cellular mixture rather than providing conventional external validation. Adding epithelial and stromal material changed both the denominator of the single‐cell fraction and the transcriptional background of the bulk sample. Plasma retained a strong patient‐level module correlation, but only five independent patients were available, and the exact inference varied by representation: the module result met the predefined exploratory criterion, whereas ProM did not. This difference indicates that the direction of the signal was reproducible, but its magnitude and inferential support were not consistent across reference models or tissue preparations.

The Plasma association with CR versus PD in the retained IMvigor210 subset weakened after adjustment for total immune expression. In repeated cross‐validation, adding Plasma reduced ROC AUC and increased Brier error in both model comparisons. The small increase in average precision without tumor mutational burden was not retained in the tumor‐mutational‐burden models, so the metrics did not support a consistent gain. GSE176307 also separated prognosis from treatment response; several immune scores were associated with longer survival in unadjusted models without showing objective‐response discrimination. Because the survival models were unadjusted, clinical differences between patients may partly account for those associations.

The secondary analyses examined separate predefined programs in BCG‐treated non–muscle‐invasive disease, longitudinal tissue samples, peripheral blood, and an additional single‐cell cohort. None of the clinical associations survived correction within its analysis family. Differences in program definitions, biological compartments, and endpoints limit comparison across these datasets; the results describe related immune processes in distinct settings rather than replication of the primary state modules.

The LY6E analysis addressed a complementary question: whether a tumor‐upregulated gene expressed in epithelial cells contributed to cancer‐cell phenotypes. The selection criteria favored a perturbable gene with tumor‐associated expression, epithelial compatibility, external expression support, and an adverse clinical direction. In 5637 and J82 cells, two nonoverlapping shRNAs produced concordant reductions in short‐term growth, colony formation, and migration while increasing apoptosis. Re‐expression of an shRNA‐resistant LY6E construct restored both transcript abundance and clonogenic growth after sh‐LY6E‐1, strengthening the link between LY6E depletion and the colony phenotype. Experimental work has independently linked tumor‐cell LY6E to impaired antitumor T‐cell responses [53], gastric cancer models provide precedent for proliferative and metastatic effects [54], and its surface localization has supported antibody‐drug‐conjugate development in solid tumors [55]. The knockdown and rescue findings extend this functional evidence to two bladder cancer cell lines. Clinical interpretation is less straightforward. The GSE13507 survival curve and adjusted estimate were nonsignificant, LY6E was expressed in both immune and epithelial cells, and IMvigor210 and GSE176307 showed opposing association directions under different response definitions. These differences may reflect cellular composition or cohort context and warrant evaluation under a common endpoint. The survival evidence also contributed to candidate selection and is therefore not an independent assessment of a prespecified gene.

The main limitation is the number of independent matched specimens. Eight CD45‐enriched pairs produce wide bootstrap intervals, and five cross‐preparation patients yield a coarse exact permutation distribution. The two single‐cell preparations had no shared donors, but the reference and paired bulk profiles came from the same study. State eligibility was fixed before the leave‐one‐patient‐out marker checks, so those checks did not independently validate the entire selection procedure. Their observed fractions depend on tissue dissociation, enrichment, and cell recovery; deposited annotations, ambient RNA, doublets, and donor‐specific expression may propagate into the modules. The IMvigor210 analysis omitted partial‐response, stable‐disease, and unevaluable cases and therefore cannot be generalized to objective response in the complete trial cohort. In the clinical cohorts, few outcome‐positive cases, correlated immune predictors, and incomplete tumor‐mutational‐burden data limit multivariable precision. Predictive performance was assessed by internal cross‐validation; external validation with the same endpoint and preprocessing pipeline remains necessary. Spatial localization, immunoglobulin clonality, protein expression, and histologic Plasma‐cell density were unavailable.

The cell experiments were limited to two bladder cancer models. Each condition was represented by three independent experiments, the perturbations were transient, and knockdown was verified at the transcript rather than protein level. The rescue experiment addressed clonogenic growth; migration and apoptosis were evaluated only after knockdown. No normal urothelial model, direct pathway assay, animal experiment, or patient‐derived validation was included. These experiments support an in vitro contribution to the measured phenotypes but leave tumor selectivity and downstream signaling unresolved. They did not test immune‐cell interactions or checkpoint‐inhibitor response.

Matched bulk RNA, unsorted single‐cell or single‐nucleus profiles, histology, and orthogonal cell‐density measurements from the same specimens could clarify how Plasma‐related transcription relates to cellular abundance and spatial organization. For LY6E, protein‐level perturbation and rescue across additional phenotypes and urothelial models would help distinguish a general effect on cell fitness from a more specific function.

## 5. Conclusion

Matched single‐cell and bulk transcriptomes recovered Plasma‐related immune abundance in urothelial carcinoma, but the score provided no consistent improvement in CR‐versus‐PD prediction in IMvigor210. LY6E knockdown and rescue supported its contribution to clonogenic growth in the tested bladder cancer cell lines.

## Author Contributions

Yitao Huang contributed to conceptualization, methodology, formal analysis, investigation, visualization, and writing – original draft. Chao Zhang contributed to methodology, software, formal analysis, data curation, visualization, and writing – original draft. Xiuzhuang Xie contributed to investigation, validation, data curation, and writing – review and editing. Xiaye Miao and Zhang Quan contributed to conceptualization, supervision, resources, project administration, and writing – review and editing. Yitao Huang, Chao Zhang, and Xiuzhuang Xie contributed equally.

## Funding

No funding was received for this manuscript.

## Disclosure

All authors reviewed and approved the final manuscript.

## Conflicts of Interest

The authors declare no conflicts of interest.

## Data Availability

The public transcriptomic datasets analyzed in this study are available from the Gene Expression Omnibus under accession numbers GSE223971, GSE176307, GSE176178, GSE244895, GSE199471, GSE145281, GSE269877, and GSE13507. TCGA‐BLCA data are available through the Genomic Data Commons, and IMvigor210 data are available through the published IMvigor210CoreBiologies data resource. Source data supporting the cell experiments and derived analyses are available from the corresponding authors upon reasonable request.
